# Association between Plasma P‐tau217 and Alzheimer's Copathology and Cognitive Decline in Parkinson's Disease

**DOI:** 10.1002/ana.78201

**Published:** 2026-03-18

**Authors:** Thomas F. Tropea, Patricia Aldea Stevenson, Matthew Flitter, David Meehan, Amanda Morris, Ming Lu, Leonardo Iaccarino, Emily C. Collins, Michael E. Hodsdon, David Irwin, Meredith Spindler, Andres Deik, Nabila Dahodwala, Katheryn A.Q. Cousins, David A. Wolk, Leslie Shaw, Daniel Weintraub, Edward B. Lee, Alice Chen‐Plotkin, Mark Mintun, Andrew Siderowf

**Affiliations:** ^1^ Department of Neurology University of Pennsylvania Philadelphia PA; ^2^ Eli Lilly and Company; ^3^ Department of Pathology and Laboratory Medicine University of Pennsylvania Philadelphia PA; ^4^ Department of Psychiatry University of Pennsylvania Philadelphia PA; ^5^ Present address: Institute for Neurodegenerative Disorders New Haven CT

## Abstract

**Objective:**

Clinically relevant Alzheimer's disease co‐pathology is common in Lewy body disorders. Plasma P‐tau217 is a sensitive biomarker for amyloid and tau pathology in Alzheimer's disease. The objective was to determine if plasma P‐tau217 associates with Alzheimer's disease co‐pathology and cognition in Lewy body disorders.

**Methods:**

Participants had (1) a clinicopathological diagnosis of Parkinson's disease or dementia with Lewy bodies in the pathology series, or (2) a clinical diagnosis of Parkinson's disease in the clinical series and were followed as part of the longitudinal, observational cohort study at the University of Pennsylvania between 2007 and 2024. Plasma concentration of P‐tau217 was measured in previously collected samples.

**Results:**

Neuropathology cases included 46 Parkinson's disease and 10 dementia with Lewy bodies, and clinical cases included 173 Parkinson's disease, and 64 controls. P‐tau217 was greater in cases with (median, 0.3 [interquartile range (IQR), 0.2–0.4]) versus without (median, 0.1 [IQR, 0.1–0.2]) Alzheimer's disease co‐pathology (*p* < 0.01) and discriminates Lewy body disorders participants with Alzheimer's disease co‐pathology (area under curve, 0.84). Parkinson's disease participants with incident cognitive impairment had greater increases in serial P‐tau217 than those who remained cognitively stable (group × time interaction β = −0.010, *p* = 0.0027). Higher baseline P‐tau217 concentrations associated with longitudinal change in dementia rating scale (group × time interaction β = −4.947, *p* = 0.0166) and higher risk for cognitive progression (hazard ratio = 1.597, *p* = 0.003).

**Interpretation:**

Plasma P‐tau217 detects Alzheimer's disease co‐pathology in Lewy body disorders and predicts cognitive decline. Future studies will evaluate associations between plasma P‐tau217 and imaging and clinical outcomes, in consideration for use of amyloid‐targeting therapies in Lewy body disorders. ANN NEUROL 2026;99:1428–1437

Lewy body disorders (LBD) include Parkinson's disease (PD) and dementia with Lewy bodies (DLB) and are defined pathologically by intraneuronal inclusions of misfolded α‐synuclein (αSyn) contained in Lewy bodies (LB).^1^, ^2^ Although αSyn pathology is the strongest neuropathological correlate of cognitive symptoms in LBD,^3^, ^4^ concomitant neurodegenerative pathology is common. At death, between 25% and 55% of LBD cases also demonstrate sufficient amyloid‐beta (Aβ) and tau pathology to warrant a secondary diagnosis of Alzheimer's disease (AD).^5^, ^6^, ^7^ Concomitant AD pathology in LBD patients associates with worse motor and cognitive symptoms and shorter survival compared to those without AD copathology.^8^, ^9^, ^10^, ^11^, ^12^, ^13^ Until recently, the ability to detect neurodegenerative pathology during life has been limited to imaging or cerebrospinal fluid (CSF) biomarkers, and therefore, the temporal association between concomitant AD pathology and the clinical syndrome in LBD remain poorly understood. Recent advances in biomarkers to measure Aβ and tau in plasma by high‐throughput immunofluorescent assays have allowed for early detection of AD pathology during life, possibly before symptom onset, that are less invasive and scalable to large populations compared to imaging or CSF tests.^14^ Measures of phosphorylated tau at threonine 217 (P‐tau217) associate with Aβ and tau in autopsy cohorts and in clinical cohorts of individuals with evidence of amyloid or tau pathology measured by positron emission tomography (PET) neuroimaging or CSF biomarkers.^15^, ^16^, ^17^, ^18^, ^19^, ^20^, ^21^, ^22^ Importantly, levels of P‐tau217 in the CSF and blood may be elevated at a lower burden of Aβ and tau pathology than P‐tau181 suggesting it is a more sensitive marker for AD pathology.^23^ Increases in P‐tau181, P‐tau217, and P‐tau231 have been observed in DLB cohorts with AD co‐pathology.^24^, ^25^, ^26^ Furthermore, levels of P‐tau217 are elevated in PD with cognitive impairment compared to PD without cognitive impairment.^21^, ^22^ The performance of plasma P‐tau217 as a prognostic marker for longitudinal outcomes in PD cohorts remains uncertain.

The objectives of this study were to: (1) compare antemortem plasma P‐tau217 levels in LBD patients with and without AD co‐pathology at autopsy; (2) to examine changes in P‐tau217 levels over time in PD patients; and (3) to evaluate the prognostic value of P‐tau217 levels for cognitive decline in PD. We hypothesized that elevated plasma P‐tau217 concentration would associate with AD copathology and with worse cognition in LBD.

## Subjects and Methods

### Study Design

These studies report on data from two cohorts at the University of Pennsylvania (UPenn). The first cohort is comprised of neuropathological data with antemortem plasma samples, while the second cohort is comprised of clinical data and plasma samples collected as part of ongoing longitudinal observational studies.

### Participants and Clinical Assessments

For pathology studies, all autopsy cases enrolled between February 1985 and July 2021 in the UPenn Center for Neurodegenerative Research brain bank were assessed and included in the analysis if they (1) had a primary clinicopathological diagnosis of PD or DLB^27^, ^28^ and (2) had available antemortem plasma samples within 5 years of death. Of 1970 accessioned cases, 56 (2.8%) met the above criteria.

For clinical cases, participants were included in the analysis if they (1) had a clinical diagnosis of PD according to established criteria^28^, ^29^ or were neurologically normal healthy controls (HC), (2) had at least one plasma sample available, and (3) had a consensus cognitive diagnosis of normal or mild cognitive impairment (MCI) at study entry. All PD cases and a subset of HC cases (based on sample availability) were included. Up to 2 additional samples (maximum of 3 samples per participant) were included for a subset of individuals (n = 48) at the subsequent visit closest to a cognitive change (from normal to MCI, normal to dementia, or MCI to dementia) as well as a set of individuals matched for disease duration at last follow up (n = 33) without cognitive change (consensus cognitive diagnosis of normal over 2–3 visits when a plasma sample was collected). Eighteen of the clinical cohort cases were also included in the pathology cohort analyses because they also had an antemortem sample measure.

Clinical assessments were performed by trained research staff. At study entry and at least at yearly intervals clinical information was obtained and Mattis Dementia Rating Scale‐2 (DRS‐2)^30^ scale was administered. A consensus cognitive diagnosis of normal, MCI, or dementia was assigned to each participant at each visit by experienced raters as previously described.^31^

This study was approved by the UPenn institutional review board and participants gave written informed consent at study enrollment and for autopsy studies, informed consent was obtained from next‐of‐kin at time of death. All procedures in these studies adhere to the tenets of the Declaration of Helsinki.

### Neuropathological Staging

Neuropathological characterization of 16 brain regions was conducted on all cases as previously described to measure tau, β‐amyloid, and αSyn pathologies.^28^, ^32^ Additionally, AD Neuropathologic Change (ADNC) score^33^, ^34^ and DLB Type (modified McKeith criteria) were assigned.^27^

### Measurement of P‐tau217

Whole blood samples were collected in EDTA‐tubes, spun‐down, and stored at −80°C.^28^ Before analysis, samples were thawed, vortexed thoroughly, and centrifuged at 16,000 × *g* to eliminate particulates. P‐tau217 concentrations were quantified using a sandwich immunoassay on the Quanterix SP‐X platform using chemiluminescent detection. This assay uses 4G10E2 antibody (specific for brain tau) for detection and IBA493 antibody (specific for phospho‐tau217) for capture. A synthetic tau peptide served as calibrator across a 7‐point calibration curve spanning the analytical measurement range (0.08–2.81U/mL). Plasma samples underwent standard 1:2 dilution in assay diluent, with each sample analyzed in duplicate. Following reagent additions and wash steps, chemiluminescence from the antigen–antibody complex was captured by the SP‐X Imager and sample concentrations determined by interpolation from the standard curve using log–log power regression with results expressed in U/mL.

### Statistical Analysis

Analyses were conducted using R Statistical Software v4.4.2,^35^ Prism (version 10.4.1 for MacOS, GraphPad Software, Boston, MA, www.graphpad.com), or Stata BE/18.5 (StataCorp. 2023. Stata Statistical Software: Release 18. College Station, TX: StataCorp), and R‐scripts or do‐files are available upon request of the corresponding author. Mann–Whitney tests, Kruskall‐Wallis tests with Dunn's test for pairwise comparisons, and multiple regression were used to compare P‐tau217 concentrations between diagnosis‐ and pathology‐ groups. The ability of plasma P‐tau217 to discriminate LBD cases with (ADNC intermediate/high) versus without (ADNC none/low) AD copathology was assessed by the area under the curve (AUC) of the receiver operating characteristic (ROC) curve. Linear mixed‐effects models^36^ were used to test for associations between (1) baseline P‐tau217 concentration and cognitive test score (DRS‐2) score over time (in years) with covariates of age at baseline, sex, number of *APOE* ε4 alleles, and baseline DRS‐2 score; and (2) association of cognitive group (converter or non‐converter based on consensus cognitive diagnosis) and P‐tau217 over time (in years) with covariates of age at baseline, sex, number of *APOE* ε4 alleles and baseline P‐tau217 concentration. Education was not included because of its limited variability in this cohort, which is generally high compared to the general population, and its lack of independent association with DRS‐2. Cases included in longitudinal DRS‐2 analyses had a minimum of 0.8 years of follow up time, which was chosen to approximate 1 year of follow‐up time. Random intercept was included in each mixed‐effects model to account for correlations among repeated measures. Cox proportional hazards models were used to assess the relationship between (1) cognitive group (converter or non‐converter based on consensus cognitive diagnosis) and risk of reaching the P‐tau217 0.21U/mL high‐confidence threshold and (2) three‐interval of P‐tau217 derived in AD based on association with Aβ PET (<0.12, 0.12–0.21, and >0.21U/mL)^37^, ^38^ and risk to a change in consensus cognitive diagnosis to MCI or dementia. The proportional hazards assumption was assessed using the Schoenfield residuals global test. All statistical tests were tested with two‐sided α of 0.05.

## Results

### Participant Cohorts and Clinical Summary

Demographic, pathological, and baseline characteristics of the cohorts are shown in Table 1 and Table S1. The pathology cohort consisted of 56 autopsies (10 DLB/46 PD) completed between August 2011 and July 2021. The average interval from plasma collection to death was 1.8 years (standard deviation [SD], 1.0; maximum [max], 3.5 years). The clinical cohort consisted of 173 PD and 64 HC participants followed between November 2007 and November 2024. Among the PD cases in the clinical cohort, 92 had a single sample, 70 had 2 samples, and 11 had 3 samples included. The frequency distribution plot of P‐tau217 is shown in Figure S1A and log transformed P‐tau217 in Figure S1B.

**TABLE 1:** Cohort Description at the Time of Plasma Sampling

	Clinical		Pathology	
	Control	PD	PD	DLB
N	64	173	46	10
Age at sample, yr	72 (4.9)	72 (7.4)^a^	77 (7.5)^b^	70 (5.6)
Disease duration at sample, yr	NA	10 (5.5)^a^	14 (5.1)^b^	5 (1.9)
Sex, n (% M)	21 (33)	108 (62)	36 (78)	10 (100)
Education, yr	16 (2.4)	16 (2.4)	16 (2.9)	15 (2.9)
Race, n (%)				
Asian	0 (0)	2 (<1)	1 (2)	0 (0)
Black	29 (45)	8 (5)	1 (2)	0 (0)
White	35 (55)	162 (94)	44 (96)	10 (100)
Unknown/not reported	0 (0)	1 (<1)	0 (0)	0 (0)
*APOE* ε4 haplotype, n (%)				
ε4/−	18 (29)	36 (21)	22 (48)	5 (50)
ε4/ε4	0 (0)	2 (1)	0 (0)	0 (0)
Baseline Cog Dx, n (%)				
Normal		120 (73)		
MCI		45 (27)		
Dementia		0 (0)		
Baseline DRS		136 (14.3)		
Age at death, yr			79 (7.5)	72 (5.7)
Sample to death interval, yr			1.9 (1.0)	1.5 (0.9)
DLB type, n (%)				
Brainstem			8 (17)	0 (0)
Transitional/limbic			18 (39)	2 (20)
Diffuse/neocortical			20 (43)	8 (80)
ADNC, n (%)				
Not			10 (22)	1 (10)
Low			20 (44)	3 (30)
Intermediate			14 (30)	6 (60)
High			2 (4)	0 (0)
AD copathology, n (% yes)^c^			16 (35)	6 (60)
P‐tau217 U/mL (med, IQR)	0.068 (0.057–0.086)	0.087^a^ (0.067–0.124)	0.154^b^ (0.075–0.252)	0.162 (0.127–0.450)
P‐tau217 thresholds^a^				
<0.12U/mL n (%)	59 (92)	124 (72)	18 (39)	2 (20)
0.12–0.21U/mL	3 (5)	31 (18)	17 (37)	5 (50)
>0.21U/mL	2 (3)	18 (10)	11 (24)	3 (30)

All values are represented as mean (standard deviation), unless otherwise specified.

^a^ First sample is used in cases where multiple measurements were collected.

^b^ Sample closest to death is used in pathology cases.

^c^ AD copathology is defined as ADNC of intermediate or high.

AD = Alzheimer's disease; ADNC = Alzheimer's Disease Neuropathological Change; DLB = dementia with Lewy bodies; DRS = Mattis Dementia Rating Scale; IQR = interquartile range; M = male; med = median; MCI = mild cognitive impairment; PD = Parkinson's disease.

### Plasma P‐tau217 Discriminates Pathology‐Confirmed LBD with AD Co‐Pathology versus without AD Co‐Pathology

**FIGURE 1:**
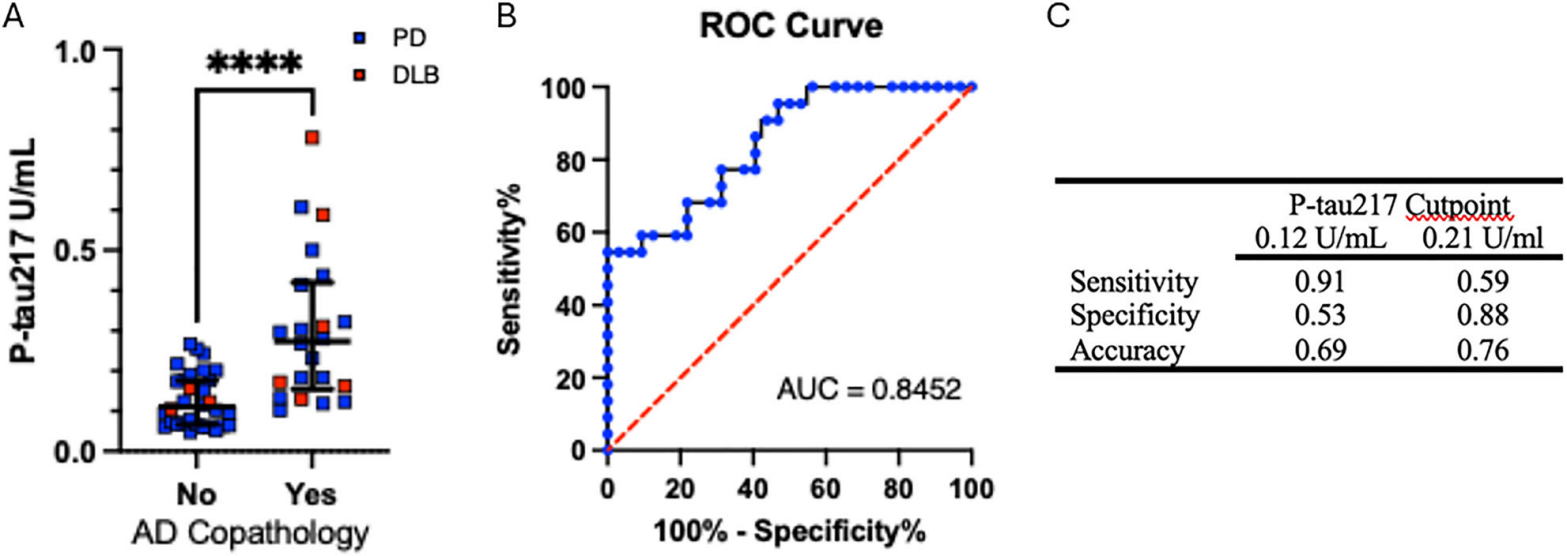
Plasma P‐tau217 in pathologically confirmed PD or dementia with DLB cases with and without AD copathology. (A) Plasma P‐tau217 concentrations (U/mL) in antemortem samples from pathologically confirmed PD and DLB in individuals with or without AD copathology (defined as AD neurological change of intermediate or high). Blue indicates PD, red indicates dementia with Lewy bodies. (B) ROC curve showing that plasma P‐tau217 concentration differentiate between cases with versus without AD copathology with AUC of 0.8452. (C) Performance of the AD‐derived plasma P‐tau217 low (0.12U/mL) and high (0.21U/mL) confidence thresholds to identify cases with versus without AD copathology. ****p < 0.0001. AD = Alzheimer's disease; AUC = area under the curve; DLB = dementia with Lewy bodies; PD = Parkinson's disease; ROC = receiver operator characteristics.

Among pathology‐confirmed cases, P‐tau217 concentrations were greater in LBD cases with (ADNC intermediate/high; median, 0.274; interquartile range [IQR], 0.155–0.421) versus those without (ADNC none/low; median, 0.110; IQR, 0.066–0.176) AD copathology (*U* = 109, *p* < 0.0001) (Fig 1A). Logistic regression covarying for age at sample, sex, sample to death interval, and *APOE* ε4 alleles also showed differences in P‐tau217 concentration between groups by AD copathology (β = 14.2607, standard error [SE] = 5.3452, *p* = 0.008). Analysis of ROC curves to determine the ability of plasma P‐tau217 to discriminate LBD individuals with versus without AD co‐pathology showed moderately high accuracy (AUC = 0.8452, 95% CI = 0.74–0.95, *p* < 0.0001) (see Fig 1). Biomarker thresholds of low (<0.12U/mL) or high (>0.21U/mL) confidence derived in AD cohorts^37^, ^38^ were evaluated for their performance in discriminating LBD with versus without AD copathology. Sensitivity, specificity, and accuracy for 0.12U/mL and 0.21U/mL thresholds are shown in Figure 1C and Figure S2.

### Plasma P‐tau217 Differs between Diagnosis Groups

P‐tau217 concentrations differ between groups (H[3] = 46.52, *p* < 0.0001) and median (IQR) are reported in Table 1. Post hoc Dunn's tests show that P‐tau217 concentration was higher the clinical PD (adj *p* = 0.0007), pathology‐confirmed PD (adj *p* < 0.0001), and the pathology‐confirmed DLB (adj *p* < 0.0001) groups versus HC. The effect remains after controlling for age, sex, and *APOE* ε4 alleles (*F*[6,279] = 9.68, *p* < 0.0001). Additionally, clinical PD differs from pathology‐confirmed PD (adj *p* = 0.0042) and pathology confirmed DLB (adj *p* = 0.0053).

### Plasma P‐tau217 Concentrations Are Greater in Individuals with More Diffuse LB, Amyloid, and Tau Pathology

**FIGURE 2:**
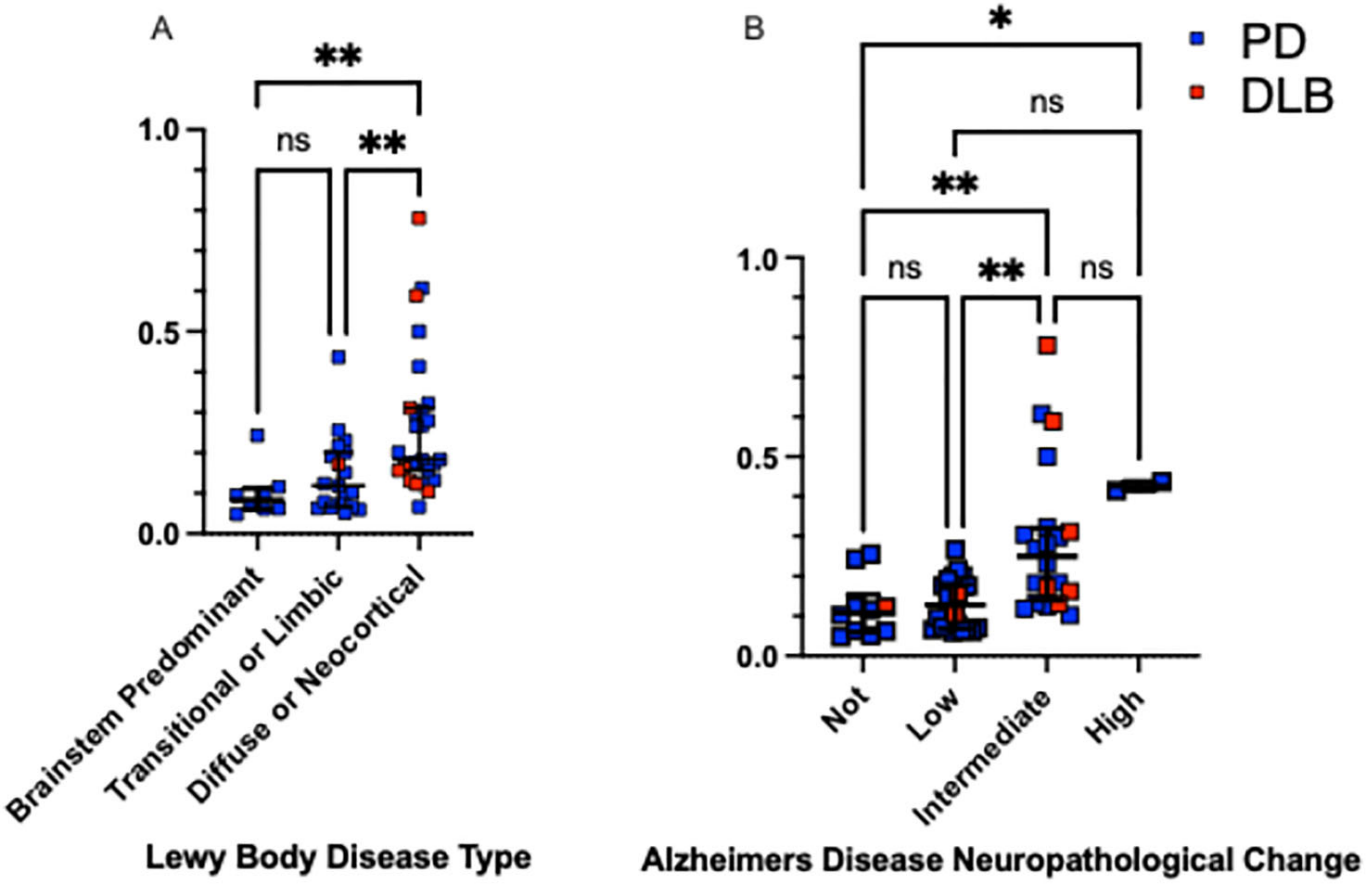
Plasma P‐tau217 concentration by (A) Lewy‐body disease modified McKeith criteria type, or (B) Alzheimer's Disease Neuropathological Change pathology groups. Each data point represents a unique participant at the sample most proximate to death Path Parkinson's disease (PD) or Path dementia with Lewy bodies (DLB). Box and whisker plot represent median P‐tau217 concentration (U/mL) +/− interquartile range. Blue indicates Parkinson's disease, red indicates DLB. *p < 0.05, **p < 0.01.

Cases with diffuse/neocortical LB pathology had higher P‐tau217 concentrations (median, 0.184; IQR, 0.15–0.31) than those with transitional/limbic (median, 0.119; IQR, 0.06–0.20) or brainstem only (median, 0.083; IQR, 0.06–0.11; group comparison H[2] = 16.47, *p* = 0.0003) (Fig 2A). P‐tau217 concentration was higher in cases with high (median, 0.426; IQR, 0.42–0.44) or intermediate (median, 0.249; IQR, 0.14–0.32) ADNC versus those without ADNC (ADNC none; median, 0.109; IQR, 0.06–0.15). ADNC intermediate was additionally higher than low (median, 0.128; IQR, 0.07–0.18; group comparison (H[3] = 19.87, *p* = 0.0002) (see Fig 2B). Cases with amyloid (Thal) A score of 3 had higher P‐tau217 concentration (median, 0.280; IQR, 0.17–0.44) than those with score of 0 (median, 0.109; IQR, 0.06–0.15) or 1 (median, 0.076; IQR, 0.07–0.18), but not 2 (median, 0.156; IQR, 0.10–0.20; group comparison H[3] = 22.52, *p* < 0.0001). P‐tau217 concentration was higher in cases with Braak (neurofibrillary tangle) B score of 3 (median, 0.415; IQR, 0.32–0.44) or 2 (median, 0.193; IQR, 0.13–0.31) versus 1 (median, 0.104; IQR, 0.07–0.18), but not 0 (median, 0.10; IQR, 0.06–0.24; group comparison H[3] = 18.83, *p* = 0.0003), although the number of cases with Braak 0 or 3 were low. P‐tau217 concentration was higher in cases with Consortium to Establish a Registry for Alzheimer's Disease (CERAD) (neuritic plaque) C score of 3 (median, 0.174; IQR, 0.15–0.46) or 2 (median, 0.289; IQR, 0.24–0.41) versus 0 (median, 0.10; IQR, 0.06–0.17), but not 1 (median 0.138; IQR, 0.10–0.19; group comparison H[3] = 23.78, *p* < 0.0001) (Figure S3).

### Serial Plasma p‐tau217 Concentrations Rise Faster in Individuals with Cognitive Worsening versus without Cognitive Worsening

**FIGURE 3:**
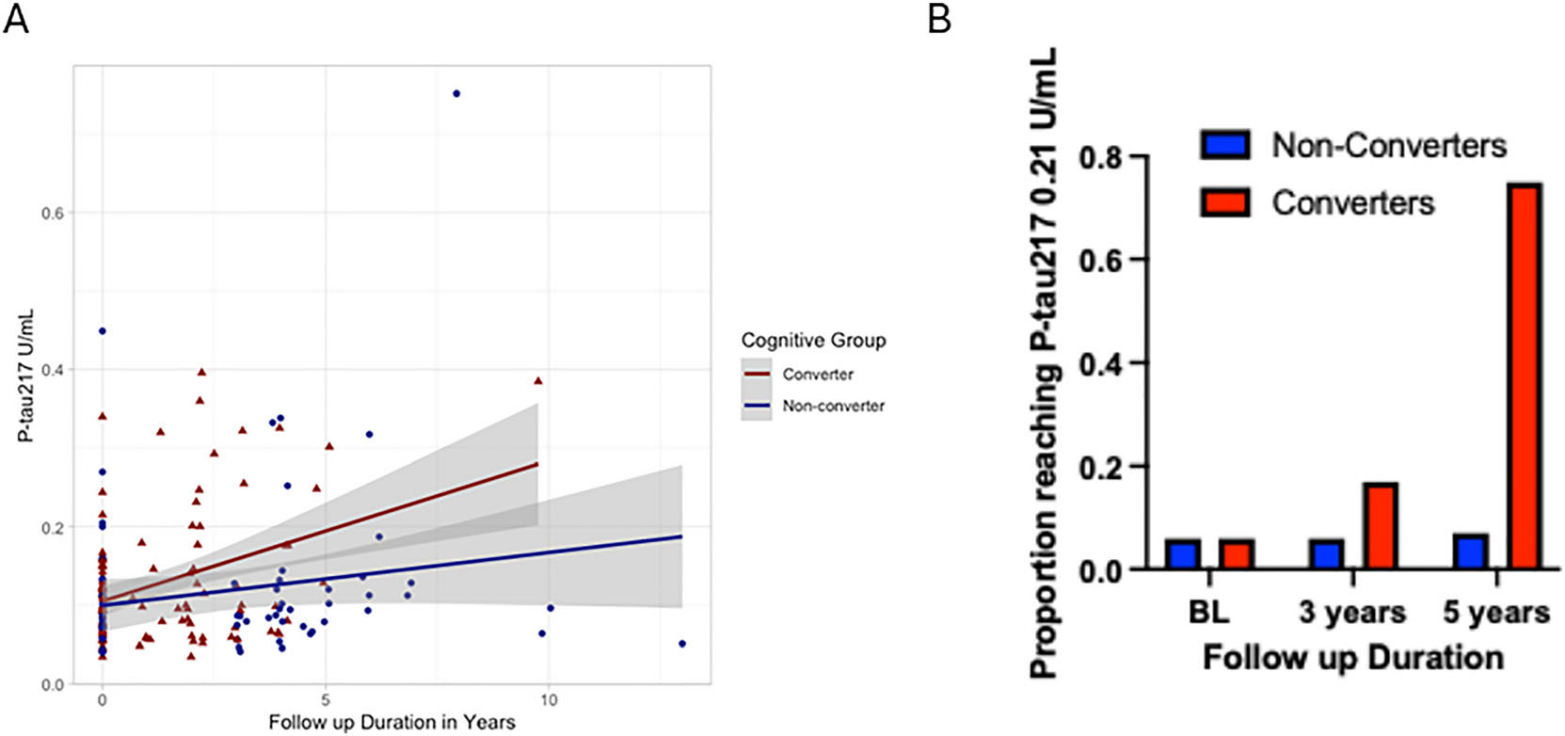
Serial P‐tau217 measures in Parkinson's disease participants with cognitive diagnosis change versus cognitively stable individuals. (A) Concentration of P‐tau217 rises faster in participants with a cognitive diagnosis change (cognitive converters, n= 48, 59%) compared to those who remain cognitively stable (cognitive non‐converter, n= 33, 41%). Participants were cognitively normal (n = 36, 75%) or had mild cognitive impairment (n = 12. 25%) at their first P‐tau217 measure, and each had 2–3 plasma P‐tau217 values at times of cognitive diagnosis determination. (B) Proportion of cognitive converters and non‐converters reaching a high‐confidence threshold of P‐tau217 (>0.21U/mL) during follow up. N of non‐converters/converters at baseline is 33/48, 3 years is 33/18, and 5 years is 14/4.

Eighty‐one cases with 1 to 13 years of follow up (mean, 3.6; SD, 2.2) had 2 (n = 70) or 3 (n = 11) serial P‐tau217 measurements selected at times of a change in cognitive diagnosis (cognitive converters, n = 48, 59%) or a set without a change in cognitive diagnosis (non‐converters, n = 33, 41%) matched for disease duration at final visit (median, 13 years; IQR, 7.8–15 in converters vs median, 13 years; IQR, 9–16 in non‐converters). At study entry, cognitive converters were normal (n = 36, 75%) or MCI (12, 25%) while all non‐converters were normal cognition at study entry. At the end of the study period n = 25 (52%) had a cognitive diagnosis of MCI while n = 23 (48%) had a cognitive diagnosis of dementia. Cognitive converters were older (mean, 71.9; SD, 6.5) than non‐converters (mean, 66.5; SD, 8.1; *p* = 0.0026) and had a longer disease duration at baseline (converters mean, 10.0; SD, 5.3; and non‐converters mean, 7.8; SD, 4.7). Cognitive converters had a faster rise in serial P‐tau217 concentration over time than non‐converters (group × time interaction term β = −0.010, SE = 0.003, *p* = 0.0027) (Fig 3A and Fig S4), although the absolute change from first to last sample did not differ (median, 0.02; IQR, −0.02 to 0.1 in converters vs median, 0.02; IQR, −0.001 to 0.03 in non‐converters). At baseline, 6% of cognitive converters (n = 48) and non‐converters (n = 33) had a P‐tau217 >0.21U/mL. At 3 years of follow‐up, 17% of converters (n = 18) and 6% of non‐converters (n = 33) had a P‐tau217 >0.21U/mL. At 5 years of follow up, 75% of converters (n = 4) and 7% of non‐converters (n = 14) had a P‐tau217 >0.21U/mL (see Fig 3B).

### Plasma P‐tau217 Predicts Changes in Cognitive Performance and Cognitive Diagnosis

**FIGURE 4:**
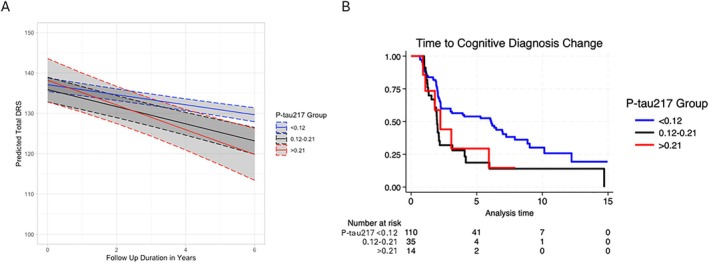
P‐tau217 concentration in Parkinson's disease participants predicts cognitive progression. (A) P‐tau217 concentration measured at study entry predicts yearly rate of change in the DRS in 154 participants followed for up to 16 years in a model correcting for age, sex, and baseline DRS score. Individuals above the low (0.12–0.21U/mL) or the high (0.21U/mL) confidence thresholds of P‐tau217 showed a greater annual decline in the DRS compared to those below the low‐confidence threshold. (B) Risk of cognitive diagnosis change differed by P‐tau217 concentration (U/mL) and individuals above the low (0.12–0.21U/mL) or the high (0.21U/mL) confidence thresholds showed greater risk compared to those below the low‐confidence threshold. DRS = Mattis Dementia Rating Scale Score.

Among the clinical PD cohort, 154 individuals had longitudinal DRS‐2 assessments for up to 16 years (mean, 5.3; SD, 3.4). The number of individuals by their longest duration of follow up is shown in Figure S5. A linear mixed effects model was used to assess the relationship between baseline P‐tau217 concentration and change in DRS‐2 score over time, adjusting for age, sex, *APOE* E4 alleles, and baseline DRS‐2 score. P‐tau217 had a significant effect on DRS‐2 over time (β = −4.947, SE = 2.060, *p* = 0.0166), with higher concentration of P‐tau217 associating with faster rate of decline in DRS‐2 (Fig 4A showing predicted yearly DRS‐2 values from the linear mixed effects model across 3 P‐tau217 groups by cut‐off). Cox proportional hazards models were used to assess the relationship between a 3‐interval P‐tau217 cut‐off (<0.12, between 0.12–0.21, and >0.21U/mL) and risk of a change in cognitive consensus diagnosis (normal to MCI, normal to dementia, or MCI to dementia). Higher P‐tau217 was associated with a greater risk of a cognitive diagnosis change when covarying for age, sex, and *APOE* ε4 alleles (HR = 1.489, SE = 0.265, *p* = 0.025) (see Fig. 4B).

## Discussion

Plasma biomarkers are quickly becoming established as diagnostic markers in AD, however, fewer studies have examined these biomarkers in PD and DLB. Preliminary evidence shows a nuanced association between P‐tau217 (or other P‐tau isoforms, including P‐Tau231 and P‐tau181) and AD neuropathology in PD that differs from AD cohorts and may differ between PD and DLB. Therefore, we leveraged a neuropathologically characterized cohort with antemortem plasma samples and a deeply characterized longitudinal clinical cohort at UPenn to examine the performance of plasma P‐tau217 to detect AD copathology and to predict cognitive decline in PD. Our findings demonstrate that plasma P‐tau217 is elevated in LBD cases with AD copathology compared to those without AD copathology. Additionally, P‐tau217 in plasma from PD collected earlier in the course of disease (10 years in clinical vs 14 years in pathology PD) was also observed to be elevated compared to HCs, but lower than pathology cases. Furthermore, we find that P‐tau217 concentration rises more rapidly in individuals with cognitive progression than in those that remain cognitively stable. Finally, in longitudinally followed individuals with PD, a single P‐tau217 measurement predicted the rate of cognitive worsening over an average of 5 years of follow up and a higher risk of cognitive diagnosis change. These findings demonstrate that plasma P‐tau217 could be a sensitive biomarker for AD pathology in PD and that P‐tau217 in mid‐stage PD predicts cognitive progression.

In AD, P‐tau isoforms associate with Aβ and tau pathology and are detectable many years before onset of symptoms.^17^, ^19^, ^20^, ^39^, ^40^, ^41^, ^42^ Yet, in cohorts comparing AD and LBD, the frequency of abnormal Aβ and tau biomarkers increases with disease duration, and changes tend to be lower in magnitude.^24^, ^25^, ^43^, ^44^ This mimics neuropathology studies where AD copathology occurs in only approximately 30–50% of LBD and tends to have lower ADNC (intermediate > high). The ADNC in this cohort of PD/DLB is overwhelmingly intermediate (35% of cases) compared to high (4%). Additionally, we see differences in P‐tau217 levels at lower relative burden of tau and neuritic plaque burden (P‐tau217 concentration is greater in Braak score or CERAD >= 2) than amyloid burden (only elevated at “A” Stage 3), suggesting that P‐tau217 may be sensitive to an earlier phase of tau compared to Aβ pathology in LBD. Yet, these findings align with a recent report showing a linear association between P‐tau217 levels and burden of semi‐quantitative tangle pathology.^45^ In that report, linear associations between P‐tau217 and burden of plaque pathology was also reported, yet increases in P‐tau217 are most pronounced only at the highest plaque burden, whereas tangle burden appears to increase linearly with elevations observed at lower relative pathology burden. Additionally, differences in biomarker measures have emerged between PD versus DLB. Notably, in one pathologically confirmed and one clinical PD study, there was no association between P‐tau181^24^ or P‐tau231^25^ and co‐occurring AD. Although in DLB, strong associations of P‐tau181 and P‐tau231 with co‐occurring AD detected by PET imaging or CSF biomarker were demonstrated.^25^, ^26^, ^39^ In these studies, the antemortem DLB cases have a far shorter disease duration than PD cohorts, and biomarker differences may arise because of the more rapid development of AD copathology in DLB as compared to PD.

The association between AD copathology and cognitive progression in PD is well established.^1^, ^2^, ^3^, ^46^ Recent reports have shown that levels of P‐tau217 increase in PD with cognitive impairment compared to PD without cognitive impairment.^21^, ^22^ Here, we show that plasma P‐tau217 increases throughout the course of PD and the increase is greater in cognitive converters, mimicking the years before cognitive impairment in AD. In this sense, cognitively normal PD with AD biomarker changes could be considered preclinical AD as well.^47^ Elevations in P‐tau217, which appear to be sensitive to early changes in AD copathology burden, reflect the role of underlying AD pathology on cognitive progression. AD copathology in PD may also have a greater clinical impact in PD because of synergistic effects between αSyn and AD pathologies.^48^, ^49^

There are some important caveats to consider when interpreting these data. For one, the limited sample size of the pathology and serial sample cohorts limits further analysis, particularly in evaluating associations between P‐tau217 and regional pathology. However, the inclusion of these extremely rare plasma‐pathology and serial plasma cases in longitudinal studies is a strength of this study. An additional limitation is the use of AD‐defined p‐tau217 thresholds that are not LBD‐specific. In the absence of a pathology confirmed validation cohort, we chose to apply thresholds that were previously validated using amyloid PET in AD cohorts as standard of truth. The lack of CSF or imaging biomarkers is an additional caveat. Finally, the HC cohort in this study is reflective of the regional population in the UPenn with greater racial diversity than is seen in the PD or DLB pathology or clinical cohorts. This may have an important effect on plasma P‐tau217 concentration as has been shown for other AD biomarkers.

## Conclusion

Plasma P‐tau217 concentrations are elevated in pathology confirmed PD and DLB that also harbor AD copathology. In longitudinal studies, increases in P‐tau217 concentration are observed in PD with cognitive progression, and a single P‐tau217 value predicts future cognitive decline. Taken together, these findings indicate that P‐tau217 is a promising biomarker to detect AD copathology and future cognitive impairment in PD and could help select participants for disease‐modifying clinical trials of amyloid‐targeting therapies.

## Author Contributions

TFT, LI, ML, ACP, MM, AS contributed to the conception and design of the study. TFT, AM, ML, LI, ECC, MED, DI, ME, AD, ND, KAQC, DAW, LS, DW, EBL, ACP, AS contributed to the acquisition and analysis of the data. TFT, AM, ML, AS contributed to drafting the text or preparing the figures.

## Potential Conflicts of Interest

P.A.S., M.F., D.M., A.M., M.L., L.I., E.C.C., M.E.H., and M.M. are employees of Eli Lilly and Company, which conducted the P‐Tau217 analyses. The remaining authors have nothing to report.

## Supporting information


**Figure S1.** Frequency distribution of (A) plasma P‐tau217 values or (B) log‐transformed P‐tau217 values in 393 unique samples from 293 participants.

**Figure S2.** Density plots (left) and relative receiver operator characteristics curve (right) of pathology confirmed Lewy body disease cases (*N* = 56) below (designated as 0) or above (designated as 1) the predefined (A) low (0.21 U/mL) or (B) high (0.21) confidence thresholds of P‐tau217 concentration. In the density plots, the heavy vertical line denotes the threshold of P‐tau217 concentration.

**Figure S3.** P‐tau217 concentration by (A) amyloid (thal) 0–3 score, (B) tau Braak 0–3 score, or (C) CERAD score. Blue indicates Parkinson's disease, red indicates dementia with Lewy bodies. * = *p* < 0.05, ** = *p* < 0.01, *** = *p* < 0.001, **** = *p* < 0.0001. CERAD, Consortium to Establish a Registry for Alzheimer's Disease.

**Figure S4.** Spaghetti plot of serial P‐tau217 measures in Parkinsons's disease participants with cognitive diagnosis change (cognitive converters, *N* = 48, 59%) versus cognitively stable (cognitive non‐converter, *N* = 33, 41%). Participants were cognitively normal (*N* = 36, 75%) or had mild cognitive impairment (*N* = 12. 25%) at their first P‐tau217 measure, and each had 2–3 plasma P‐tau217 values at times of cognitive diagnosis determination.

**Figure S5.** Number of individuals followed at the longest duration of follow up in the Parkinson's disease longitudinal clinical cohort.

**Table S1.** Neuropathological features of the postmortem cohort.

## Data Availability

The data and statistical scripts that support the findings of this study are available from the corresponding author on reasonable request.
