# Supplementary figures and images for: Association between Plasma P‐tau217 and Alzheimer's Copathology and Cognitive Decline in Parkinson's Disease
Source: Ann Neurol. 2026 Mar 18;99(6):1428–37. doi: 10.1002/ana.78201 (PMC13206273; doi:10.1002/ana.78201)

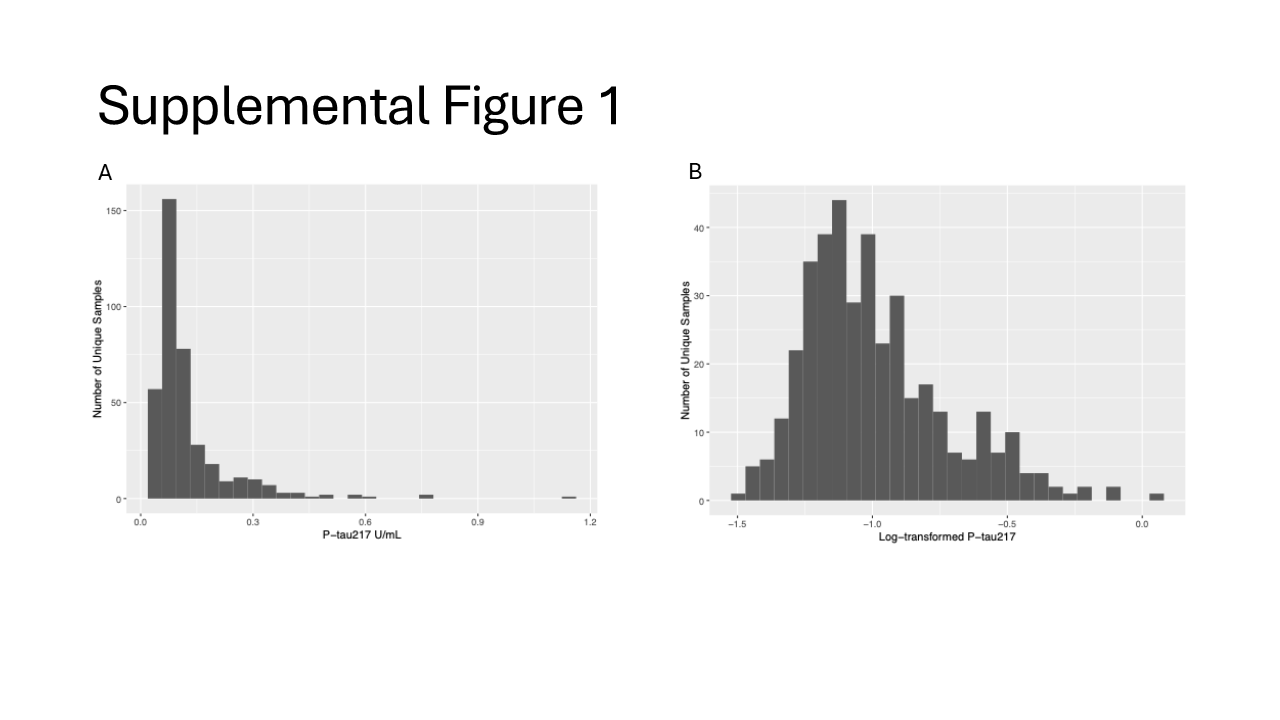

Supplement: Supplementary file 1 — Figure S1. Frequency distribution of (A) plasma P‐tau217 values or (B) log‐transformed P‐tau217 values in 393 unique samples from 293 participants. [file ANA-99-1428-s001.png]

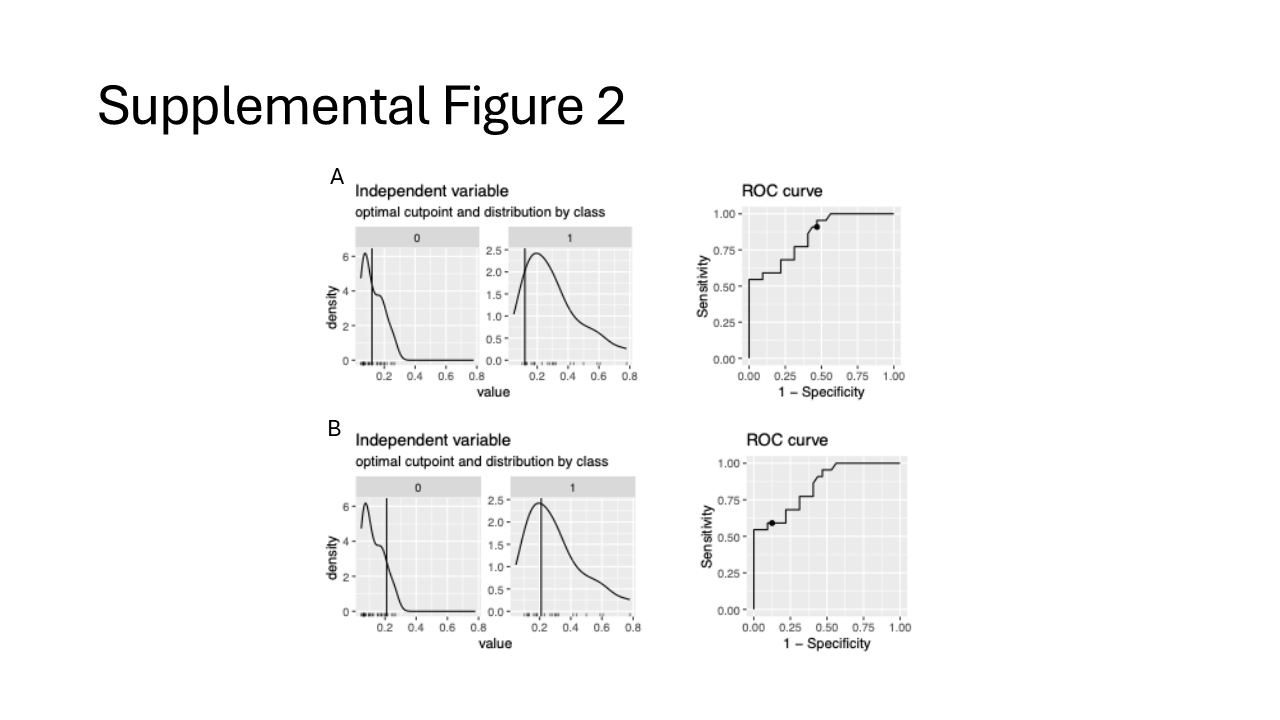

Supplement: Supplementary file 2 — Figure S2. Density plots (left) and relative receiver operator characteristics curve (right) of pathology confirmed Lewy body disease cases (N = 56) below (designated as 0) or above (designated as 1) the predefined (A) low (0.21 U/mL) or (B) high (0.21) confidence thresholds of P‐tau217 concentration. In the density plots, the heavy vertical line denotes the threshold of P‐tau217 concentration. [file ANA-99-1428-s002.png]

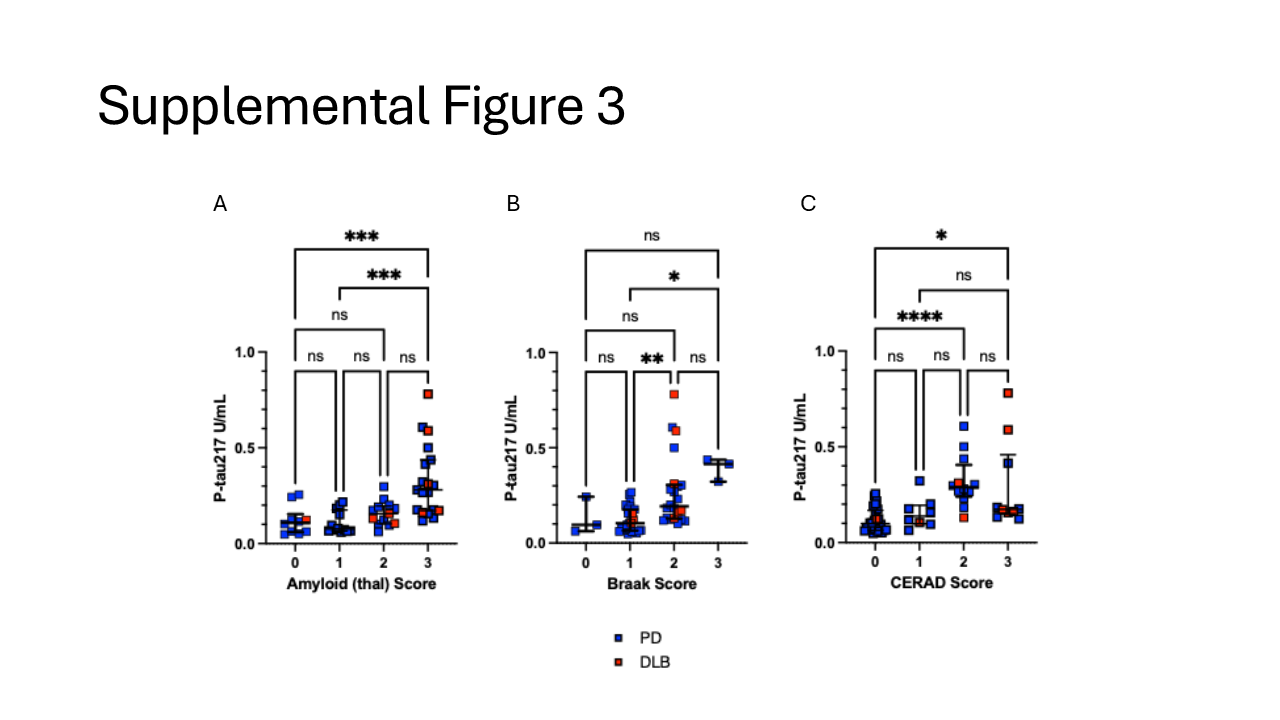

Supplement: Supplementary file 3 — Figure S3. P‐tau217 concentration by (A) amyloid (thal) 0–3 score, (B) tau Braak 0–3 score, or (C) CERAD score. Blue indicates Parkinson's disease, red indicates dementia with Lewy bodies. * = p < 0.05, ** = p < 0.01, *** = p < 0.001, **** = p < 0.0001. CERAD, Consortium to Establish a Registry for Alzheimer's Disease. [file ANA-99-1428-s004.png]

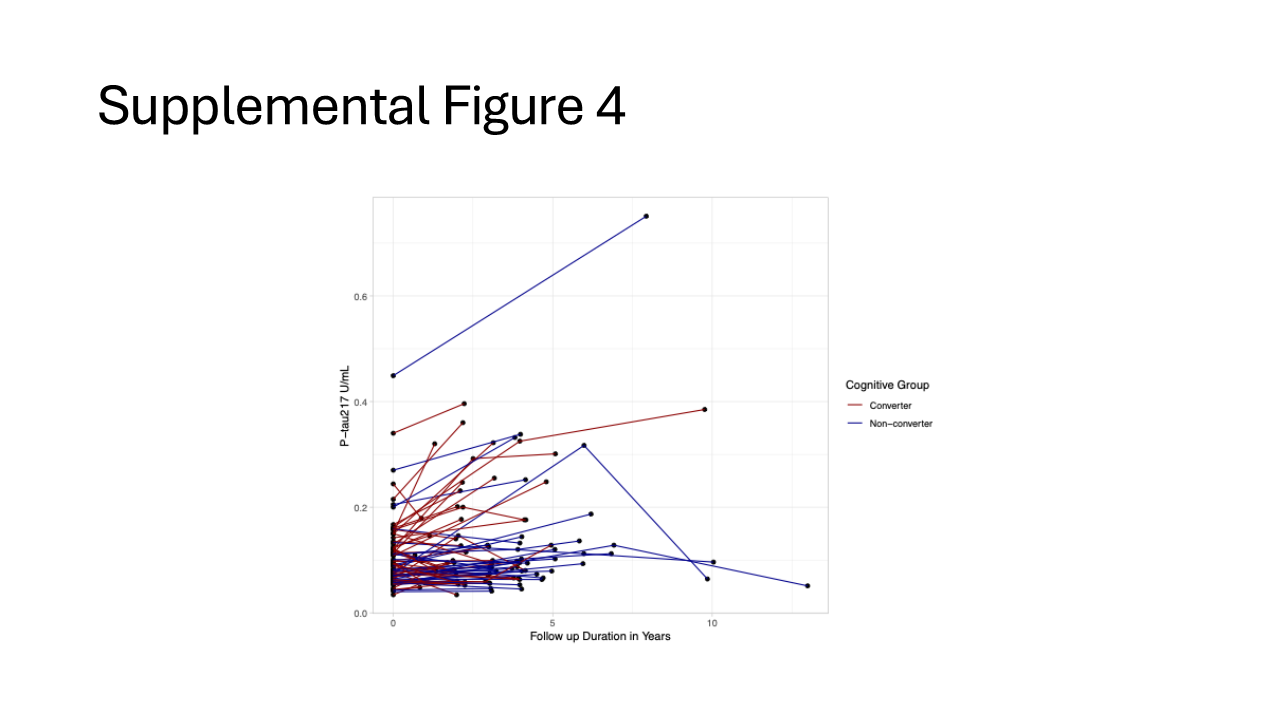

Supplement: Supplementary file 4 — Figure S4. Spaghetti plot of serial P‐tau217 measures in Parkinsons's disease participants with cognitive diagnosis change (cognitive converters, N = 48, 59%) versus cognitively stable (cognitive non‐converter, N = 33, 41%). Participants were cognitively normal (N = 36, 75%) or had mild cognitive impairment (N = 12. 25%) at their first P‐tau217 measure, and each had 2–3 plasma P‐tau217 values at times of cognitive diagnosis determination. [file ANA-99-1428-s003.png]

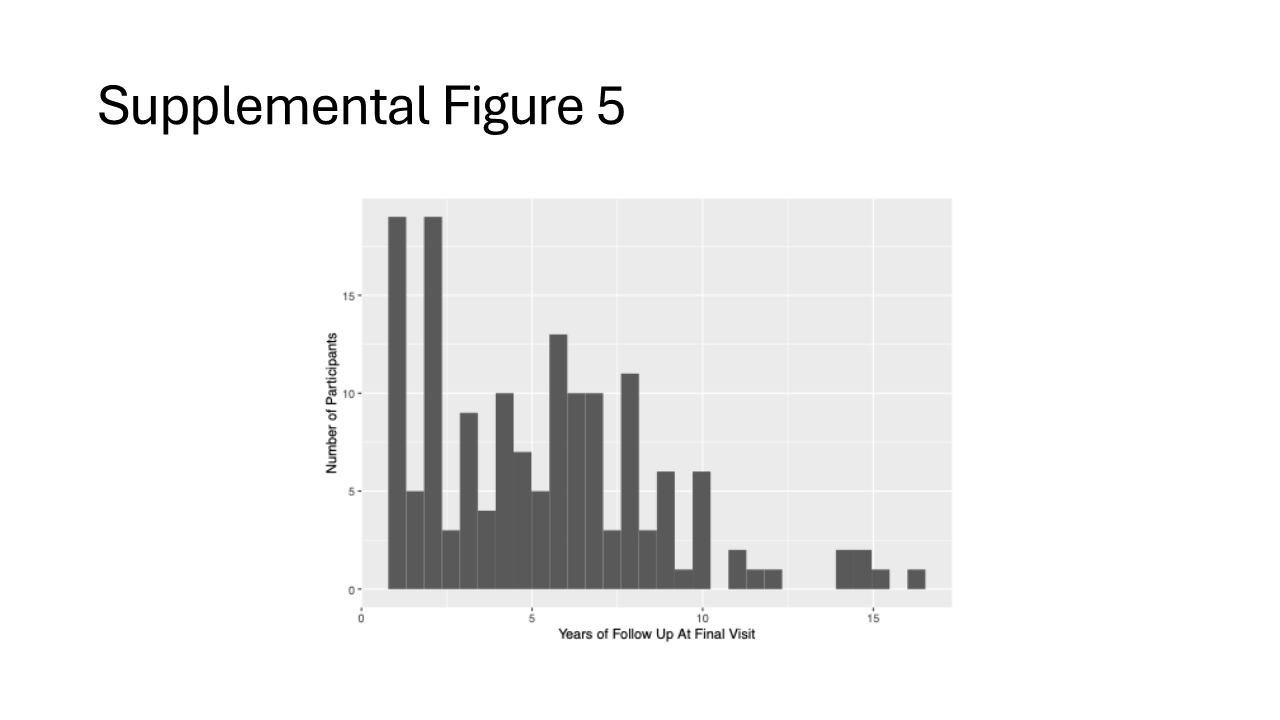

Supplement: Supplementary file 5 — Figure S5. Number of individuals followed at the longest duration of follow up in the Parkinson's disease longitudinal clinical cohort. [file ANA-99-1428-s005.png]
