# Supplementary material for: Association between Plasma P‐tau217 and Alzheimer's Copathology and Cognitive Decline in Parkinson's Disease
Source: Ann Neurol. 2026 Mar 18;99(6):1428–37. doi: 10.1002/ana.78201 (PMC13206273; doi:10.1002/ana.78201)
Supplement: Supplementary file 6 — Table S1. Neuropathological features of the postmortem cohort. [file ANA-99-1428-s006.docx]

**Supplement to** “Association between plasma P-tau217 and Alzheimer’s copathology and cognitive decline in Parkinson’s disease”

**Authors**: Thomas F Tropea DO,^1*^ Patricia Aldea Stevenson,^2^ Matthew Flitter,^2^ David Meehan,^2^ Amanda Morris,^2^ Ming Lu MD MS MPH,^2^ Leonardo Iaccarino PhD,^2^ Emily C Collins PhD, ^2^ Michael Hodsdon MD PhD,^2^ David Irwin MD,^1^ Meredith Spindler MD,^1^ Andres Deik MD,^1^ Nabila Dahodwala MD,^1^ Kathryn AQ Cousins PhD,^1^ David A. Wolk MD,^1^ Leslie Shaw PhD,^3^ Daniel Weintraub MD,^4^ Edward B Lee MD PhD,^3^Alice Chen-Plotkin MD,^1^ Mark Mintun MD,^2^ Andrew Siderowf MD.^1^

**Affiliation**: ^1^University of Pennsylvania, Department of Neurology, ^2^Eli Lilly and Company, ^3^University of Pennsylvania, Department of Pathology and Laboratory Medicine, ^4^University of Pennsylvania, Department of Psychiatry.

**Supplemental Figures Captions.**

**Supplemental Figure 1.** Frequency distribution of A) plasma P-tau217 values or B) log-transformed P-tau217 values in 393 unique samples from 293 participants.

**Supplemental Figure 2.** Density plots (left) and relative receiver operator characteristics curve (right) of pathology confirmed Lewy body disease cases (N=56) below (designated as 0) or above (designated as 1) the predefined A) low (0.21 U/mL) or B) high (0.21) confidence thresholds of P-tau217 concentration. In the density plots, the heavy vertical line denotes the threshold of P-tau217 concentration.

**Supplemental Figure 3**. P-tau217 concentration by A) amyloid (thal) 0-3 score, B) tau Braak 0-3 score, or C) CERAD score. Blue indicates Parkinson’s disease, red indicates dementia with Lewy bodies. *=p<0.05, **=p<0.01, ***=p<0.001, ****=p<0.0001. CERAD= Consortium to Establish a Registry for Alzheimer's Disease.

**Supplemental Figure 4**. Spaghetti plot of serial P-tau217 measures in Parkinsons’s disease participants with cognitive diagnosis change (cognitive converters, N=48, 59%) versus cognitively stable (cognitive non-converter, N=33, 41%). Participants were cognitively normal (N=36, 75%) or had mild cognitive impairment (N=12. 25%) at their first P-tau217 measure, and each had 2-3 plasma P-tau217 values at times of cognitive diagnosis determination.

**Supplemental Figure 5**. Number of individuals followed at the longest duration of follow up in the Parkinson’s disease longitudinal clinical cohort.

**Supplemental Table 1.** Neuropathological Features of the Postmortem Cohort.

| **Neuropathological Diagnosis** | **Primary** | **Secondary** | **Tertiary** |
| --- | --- | --- | --- |
| Lewy Body Disease | 56 (100) | 0 (0) | 0 (0) |
| Alzheimer’s Disease | 0 (0) | 44 (78) | 1 (2) |
| Cerebrovascular Disease | 0 (0) | 1 (2) | 4 (7) |
| Limbic-predominant age-related TDP-43 encephalopathy (LATE) | 0 (0) | 1 (2) | 8 (14) |
| Primary age-related tauopathy (PART) | 0 (0) | 6 (11) | 3 (5) |
| Argyrophilic grain disease | 0 (0) | 1 (2) | 0 (0) |
| Hippocampal sclerosis | 0 (0) | 1 (2) | 0 (0) |
| Cerebral amyloid angiopathy | 0 (0) | 0 (0) | 1 (2) |
| Chronic traumatic encephalopathy | 0 (0) | 0 (0) | 2 (4) |
| None | 0 (0) | 2 (3) | 37 (66) |
